# International Chinese students’ experiences of participating in mandala-based art therapy in Korea: a phenomenological study

**DOI:** 10.3389/fpsyg.2023.1263754

**Published:** 2023-10-19

**Authors:** Ya Nan Mo, Kyung Soon Ko

**Affiliations:** ^1^Independent Researcher, Taiyuan, China; ^2^Department of Creative Arts Psychotherapy, Jeonju University, Jeonju, Republic of Korea

**Keywords:** international Chinese students, South Korea, mandala, art therapy, phenomenological study, study abroad

## Abstract

**Background:**

Although China and South Korea share a similar East Asian culture, owing to major social and cultural differences, international students encounter several difficulties, which can lead to various psychological problems.

**Objective:**

To explore the experiences of eight Chinese doctoral students in South Korea participating in mandala-based art therapy.

**Method:**

Data for this phenomenological study were gathered through individual interviews and visual art from April 5 to 20, 2022. The MAXQDA software program was used for data analysis.

**Results:**

The data analysis results yielded 355 codes, 53 subcategories, and 17 categories. Five final themes emerged: (a) sealed lips by others’ eyes, (b) inner exploration and outward expression, (c) healing power of the mandala, (d) filling the inner space together, and (e) opportunities to understand art therapy.

**Conclusion:**

The researchers hope that completing this intervention will enable participants to complete their studies more effectively and achieve their ideals as well as future career goals, helping them ultimately return home safely with both personal and academic growth.

## 1. Introduction

Chinese students accounted for the largest proportion of foreign students in South Korea in 2020; with 67,348 (44.2%) students, China ranks first among the 229 countries whose students come to South Korea (Ministry of Education of Korea, 2021). There are several reasons for this. First, as China and South Korea are geographically close and have a long history of cultural exchanges, it is convenient to send students to these corresponding countries (Zhang, 2015). Second, in the field of education, after the signing of the Korea–China Cultural Exchange Agreement in 1994, Chinese students began to flood South Korea. With a rapid development in China’s economy, an increasing number of students choose to study abroad (Ko and Kim, 2020).

Even though China and South Korea share a similar East Asian culture, major social and cultural differences exist. These differences cause several difficulties for international students; the pressure they experience can lead to various psychological problems (Ismail and Yang, 2003; Tian, 2020; Zhang et al., 2022). Psychosocial support programs, freely available to any student who wants them, may help address these needs.

Art therapy helps express emotions and explore needs through visual art work (Van Lith, 2016). With an art-based modality, Chinese students can express themselves fully, even if their Korean is not fluent (Yang and Lee, 2010). Mandala-based art therapy (MBAT) specifically emphasizes the integrity and unity of the human body and mind; it can coordinate and adjust human energy, while providing energy and strength (Moss, 2007). Mandala work, which harnesses the subconscious through painting, can provide a relief outlet for depression and anxiety, guiding neuro-emotions into a stable and comfortable state (Girija and Adlin, 2018).

Regarding existing research on MBAT, some studies have noted its positive impact on adolescent mental health, allowing teenagers in crisis to express their inner repression and maintain psychological stability, so as to develop and show their inner potential (Park, 2014). Other studies have focused on populations of different ages, such as children, adults, and older adults, with most of the outcomes being related to mood, stress, and mental health (Choi and Choi, 2012). Moreover, a few studies have shown a positive impact of MBAT on specific groups such as people with schizophrenia (Lee and Byeongkug, 2010) and female victims of domestic violence. Although the use of mandalas in art therapy has been explored, these studies have utilized quantitative data, and none offer qualitative information focusing on Chinese students studying in South Korea.

### 1.1. Chinese students in South Korea

In recent years, the number of foreign students in South Korea has continued to grow along with the globalization trend of the education field. At the same time, the number of Chinese students studying abroad has also increased (Lan, 2021), with China accounting for the largest proportion of foreign students coming to South Korea (Ministry of Education of Korea, 2021).

Studies show that Chinese students in South Korea often experience stress owing to key differences in communication and interpersonal relationships (Ko and Kim, 2020). In terms of interpersonal relationships, the Chinese are more group-oriented and interdependent than South Koreans (Kim, 2015). There is also a natural language barrier (Zhao and Lee, 2018). These differences may cause Chinese students studying in South Korea to experience cultural, academic, and interpersonal difficulties. Chinese international students often experience various forms of depression and anxiety owing to differential treatment and cultural shock (Jin et al., 2022); thus, psychological and emotional supportive interventions could help prevent and address such issues.

### 1.2. MBAT

Art therapy is a way of using the fine arts, a nonverbal medium, to express personal growth, insight, and change. The special process of creating art works can help visually express human images and become an appropriate means to express individual minds. Participants can then go on to recognize their inner experiences and perceptions, connect their inner world with the external world, and work with the inner self to help discover and confirm relationships with the environment, society, and others (Judith, 1998; Malchiodi, 2006).

The purpose of group art therapy is to collectively create iconic art works, share feelings, and establish a sense of intimacy. The goal is to enable participants to easily express emotions, problems, and conflicts in a nonverbal manner, harmoniously resolving psychological and emotional issues and thus alleviating painful emotions or experiences (Malchiodi, 2006; Yalom, 2020). Such a group is not a simple collection of individuals but a dynamic gathering that seeks to solve inner problems through interaction (Jun and Choi, 1998).

The word *mandala* originates from Sanskrit in ancient India, referring to all round objects with centers in nature; it implies a “round wheel” and “round phase.” A mandala can thus be defined as the center and essence of perfection (Jung, 1973; Stout, 2014). According to Jung (1973), mandala painting could capture a change from an irregular and chaotic state to a simple and clear one. The archetype within the mandala emits a visual image to convey information when it contradicts or splits within itself. When this kind of image is expressed through art, it is easy to grasp., especially when art psychotherapy is practiced. In this way, people can better understand their internal situation by integrating their painting with themselves. A mandala is basically a “circle” image, which makes people feel relaxed and happy, with a sense of balance and order (Jung, 1973).

In art therapy, the process of painting a mandala allowing the practitioner to experience an interaction with the universe, and to discover one’s center and source. It allows people to seek stability, reconnect their inner self with order, and attain balance (Moss, 2007; Girija and Adlin, 2018). MBAT is not only a treatment for mental diseases but also a preventive intervention that can build psychological preparation and strength to accept crises and difficulties. As a psychoeducational tool, it can bring mental quietude, boosting concentration and creativity (Kim et al., 2018).

Using art-based modalities makes it easier for individuals to express themselves nonverbally, thus helping alleviate emotional crises quickly, even with language barriers (Van Lith, 2016). As group therapy, the group members can build awareness of others by focusing on themselves. Although work is done as a group, the mandala represents an inherently personalized process of growth and self-integration (Cha, 2012). MBAT allows practitioners to freely express themselves and experience positive interactions with group members through various media (Malchiodi, 2006), with this creative work helping mitigate and resolve conflicts or pressures (Judith, 1998; Van Lith, 2016). Within the group, MBAT can improve individuals’ emotional intelligence and social skills (Kim and Chun, 2015), allowing the practitioners to communicate smoothly; they learn to understand, care for, and accept each other to improve their interpersonal relationships (Kim, 2012). Studies have also shown that MBAT can improve the social adaptability of teenagers, helping them build self-expression, self-understanding, and mutual relations (Jun and Choi, 1998).

Although mandala is familiar with the Chinese culture, there are very few studies on mandala use by Chinese students. As previously mentioned, Chinese students studying in South Korea often feel stressed owing to key differences in communication and interpersonal relationships from their native culture (Kim, 2015). Therefore, it is hoped that the use of MBAT can improve the social adaptability of Korean Chinese students, enabling them to find channels for self-expression and self-understanding, and eventually improve their interpersonal relationships. Alongside the established benefits of using MBAT, our findings may prove significant in assessing whether MBAT can help participants gain psychological security, understand their inner world, and perceive stress; these are all the critical skills required by Chinese students studying in South Korea to improve their ability to cope with stress and help them decompress. In this study, therefore, we explored the experiences of Chinese students participating in MBAT in South Korea. The research question was as follows: What are international Chinese students’ experiences of participating in MBAT in South Korea?

## 2. Research methods

### 2.1. Phenomenological study

In this phenomenological study, we used a descriptive research method, which helps reveal the essential meaning behind human experiences (Van Manen, 1990; Moustakas, 1994; Giorgi, 2009). A phenomenological study attempts to understand the life experienced by human beings in the real world, and lived experience research has been widely used in various fields of the humanities and social sciences (Paley, 2016). People are individual, subjective beings, whose responses to an event are different; these distinct responses to noumena themselves act as stimulus cues (Frederick, 2005). Phenomenology involves observing the exact phenomenon, describing the participants’ experiences in an original way, understanding the circumstances, and exploring their meaning. It revolves around subjective experiences and finding elements of objective experiences common to individuals (Lewis, 2015). Accordingly, phenomenological research was appropriate for this study, which aimed to explore the lived experiences of Chinese students in South Korea.

### 2.2. Recruitment of participants

Participants—Chinese students currently studying in South Korea—were recruited by snowball sampling. This is the method of choice when conducting research with people or organizations within interactive networks or when respondents are limited and difficult to find (Yu, 2021). The selection criteria for Chinese students were as follows: (a) born and brought up in China, (b) studying for a degree in South Korea, and (c) voluntary participation. To recruit participants, the researchers provided the study instructions through WeChat, an instant messaging software mainly used by Chinese students. The sample consisted of eight participants recruited at the MBAT program at J University in South Korea from April 5 to 20, 2022. All students participated voluntarily and without a reward. All eight participants (two women, six men) were doctoral candidates in South Korea. Their ages ranged from 26 to 38 years (mean = 31) and their duration of residence was 22 to 34 months (mean = 26 months). All participants were given pseudonyms to protect their privacy (Table 1). It should be mentioned that the participants included four music therapy students and two art therapy students. These students, who had been involved in music or art therapy once or twice but had never participated in MBAT, thus developed interest in MBAT.

**Table 1:** Participants’ basic information.

Pseudonym	Gender	Age (years)	Major	Duration of residence in South Korea (months)
Yun	Man	38	Music therapy	22
Gong	Woman	32	Music therapy	22
Liu	Man	32	Music therapy	34
Li	Man	30	Music therapy	28
Shi	Man	31	Art therapy	28
Han	Man	26	Art therapy	28
Xie	Man	32	Physical education	22
Wang	Woman	30	Physical education	22

### 2.3. Intervention

Eight Chinese international students living in South Korea completed a group art therapy program assisted by experienced South Korean art therapists. The therapy program was conducted in the fine arts treatment room of J University for eight sessions. A certified art therapist was fully involved during all eight sessions. The early stage of the program (sessions 1–2) was meant for the participants to release tension and form a sense of intimacy; the middle stage (sessions 3–6) was geared toward self-expression and exploration; the final stage (sessions 7–8) was for self-integration and concluding the activities. Each session of the treatment plan lasted 120 min (this length was based on interpretation time and group size) and consisted of three stages: introduction (20 min), task and activity (60 min), and organizational and verbal sharing (40 min). Activities included enhancing the sense of collective belonging and cohesion, exploration and expression of safe areas, exploring identity through past and present life, expressing the core themes and emotions of life in the present, exploring and expressing current life stress and coping methods, expressing and integrating their own resources and shadows, identifying new identities through life goals and achievement process, and the internalization and integration of the overall treatment process (Table 2). No participants withdrew throughout the study.

**Table 2:** MBAT program: themes and directives for each of the eight sessions.

Session #	Themes and directives
1	**Dear name**
	Self-introduction of one’s name by image or symbol.
2	**Safe space**
	By completing the activity of “I in the Eyes of Others,” we dispel awkwardness and enhance the cohesion of the group. Explore and visualize your own safety zone using the five senses.
3	**The time of life**
	Set the circle as the clock of life, explore, and express the important moments in life.
4	**The wheel of life**
	Explore and express the core themes and feelings of your present life in the center of the circle.
5	**Circular cuboid**
	Current pressures are explored within the circular box and represented in a collage. Explore the coping methods you could use to relieve these sources of stress outside the round box, and express them with a collage.
6	**Light and shadow**
	Explore your own resources and shadows, and the integration and expression of resources and shadows.
7	**The four seasons of life and the trees of growth**
	Looking back on the first six mandala sessions, show how you have achieved life goals and what you have achieved.
8	**Insight and integration**
	Looking back on the first seven mandala sessions, what do you see? How would you integrate this?

### 2.4. Data collection

Data were gathered via individual semi-structured interviews, visual art works, and researcher notes. The researchers conducted 90-min individual semi-structured interviews with each participant after they completed eight sessions (Table 3). This interview was conducted in a quiet space of the art therapy room from June to July 2022. Interviews were conducted in Chinese (participants’ native language), which supported a rich and deep verbal sharing, and were videotaped with participants’ permission. The 64 visual art works generated during these sessions were also collected (eight participants over eight sessions, one mandala per participant per session), and were used to support the lived experiences that participants shared during sessions.

**Table 3:** Five themes and 17 supporting categories derived from the interview data analysis.

Themes (5)	Categories (17)
Sealed lips by others’ eyes	Worried about the prejudices
	Afraid of self-expression and openness
Inner exploration and outward expression	Focusing on the self
	Understanding myself
	Channels of self-expression
Healing power of the mandala	Relief in the mandala
	Safe place in the mandala
	Leisure in the mandala
	Satisfaction in the mandala
	Confidence in the mandala
	Courage in the mandala
	Vision in the mandala
Filling the inner space together	Communicate sincerely with group members in the same situation
	Emotional support from group members in the same cultural background
	Interpersonal interaction activity in compatriot group members
Opportunities to understand art therapy	Improve expression skills through unfamiliar opportunities
	An insight into art therapy and mandala

### 2.5. Triangulation

Member-checking was conducted to ensure an accurate understanding of interview data (Mabry, 1998). The primary researcher transcribed interviews as soon as possible to confirm accuracy and to allow participants to conduct a critical review and modify or delete their responses as needed. Peer debriefing (Lincoln and Guba, 1985; Spall, 1998; Janesick, 2004; Lewis, 2015) for data analysis took place throughout the study, from the beginning of sharing the original transcription with codes, to ensure feasibility and consistency.

### 2.6. Researchers’ reflection

The researchers have studied expressive arts therapy abroad, equipping them with great sensitivity regarding this topic. However, to prevent previous experience from influencing data interpretation, the primary researcher bracketed her own bias and preconceived knowledge on this topic to ensure that one was explicitly aware of the influence this would have on the data analysis process and results. It brought me to a bias that art therapy students have a deeper internal reflection. As a student majoring in art therapy. As the interviews were conducted in Chinese as the native language by a researcher, translated into Korean, and then translated into English for journal publication, the researchers were meticulous in verifying their translations.

### 2.7. Data analysis

MAXQDA for qualitative data analysis was used to analyze data. The analysis process was as follows: (1) organize data to access files easily; (2) read data to get familiar with the content; (3) transfer data into the MAXQDA program, with all meaningful statements being coded; (4) conduct peer debriefing with two experienced qualitative researchers; (5) combine similar and repeated semantic units, reviewing and modifying them through peer reports; (6) repeatedly confirm the original data and ensure proposed analytical feasibility, with the subcomponents being obtained through combining semantic units, and components obtained through combining subcomponents; and (7) obtain saturated final themes, categories, and subcategories.

### 2.8. Ethical considerations

This study is part of the author’s doctoral dissertation conducted with the approval of the Jeonju University Institutional Review Board (jjIRB-220421-HR-2022-0408). All participants were given details of the research project, informed of their rights, and provided written informed consent. Participants were told they had the right to refuse or withdraw from the study at any time without fear of penalty. All information was collected with the participants’ consent, and the answers were processed anonymously.

## 3. Results

The researchers analyzed the interview transcripts, yielding 355 codes related to the research topic. As a result, five themes, 17 categories, and 53 subcategory codes were identified: (a) sealed lips by others’ eyes, (b) inner exploration and outward expression, (c) healing power of the mandala, (d) filling the inner space together, and (e) opportunities to understand art therapy. Each topic contained its own unique categories, derived from the data analysis. Table 3 lists the five themes and 17 supporting categories.

### 3.1. Theme 1: sealed lips by others’ eyes

#### 3.1.1. Worried about others’ prejudices

In any psychotherapeutic group, participants need time to get comfortable with openly sharing their true selves. This was evident in this group, with Han sharing that, at the beginning of the activity, “the members of the group were not familiar with each other” and that this fed “a sense of conflict.” Shi “regarded inner things as private” and was unwilling to share. When speaking during group activities, he could not “let go of the fear that others do not understand” and could not open up his heart.

While participant defensiveness is common in the early stages of any psychotherapeutic group (Park and Park, 2022), privacy seems to be particularly valued in Chinese culture (Wang, 2019). It was, therefore, especially important for the participants to establish trust and non-judgmental relationships, learning to value others’ views and seek group harmony.

#### 3.1.2. Afraid of self-expression and openness

Participants spoke about their fears of self-expression and openness in the group, as follows:

The challenge is to express what you do not want decisively. This is my biggest challenge and it is difficult for me. […] I refuse to share my heart with others very much. It is a big challenge to open myself up in activities. The tension when communicating with others makes the sharing process difficult. (Liu)

While it is natural for participants to avoid the pain of sharing, they must eventually overcome this obstacle to obtain a wholesome therapeutic experience and enable the same outcome for the rest of the group.

### 3.2. Theme 2: inner exploration and outward expression

#### 3.2.1. Focusing on the self

Participants discovered the value of paying attention to the present, focusing on themselves, and improving concentration through MBAT activities. Gong believed that the greatest significance of therapeutic activities was to focus on the present moment:

I think, rather than worrying too much about the future, I find it more important to enjoy the process and live in the present, which is my greatest gain in this activity. Therapy has given me a way of thinking, that is, to face my problems, not to worry about the future, not to be overwhelmed by the trauma of the past. I learned to appreciate the present.

**Figure 1:**
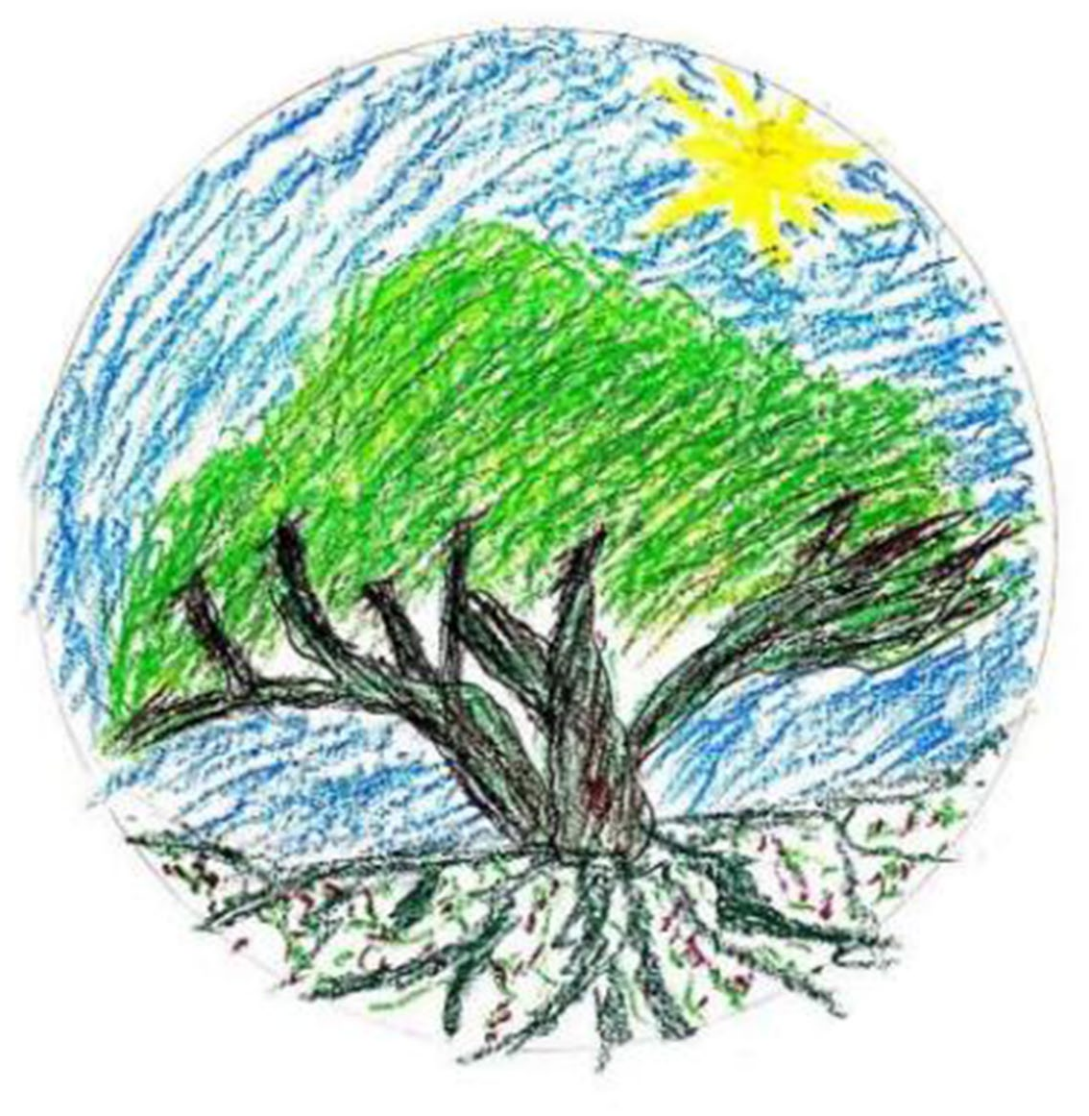
“The tree of life” by Xie. Reproduced with permission.

Wang made similar observations about concentration, saying “I also lived unconsciously and became more focused on myself.” Xie found that drawing in a circle improved his focus and attention (Figure 1):

I draw my tree of life in a circle. Without this circle, the sky part will become bigger, I might add other factors, some other embellishment. But in that case, the tree would not be so big… Based on this circle, it is visually easier for me to focus and allows me to be more focused.

#### 3.2.2. Understanding myself

**Figure 2:**
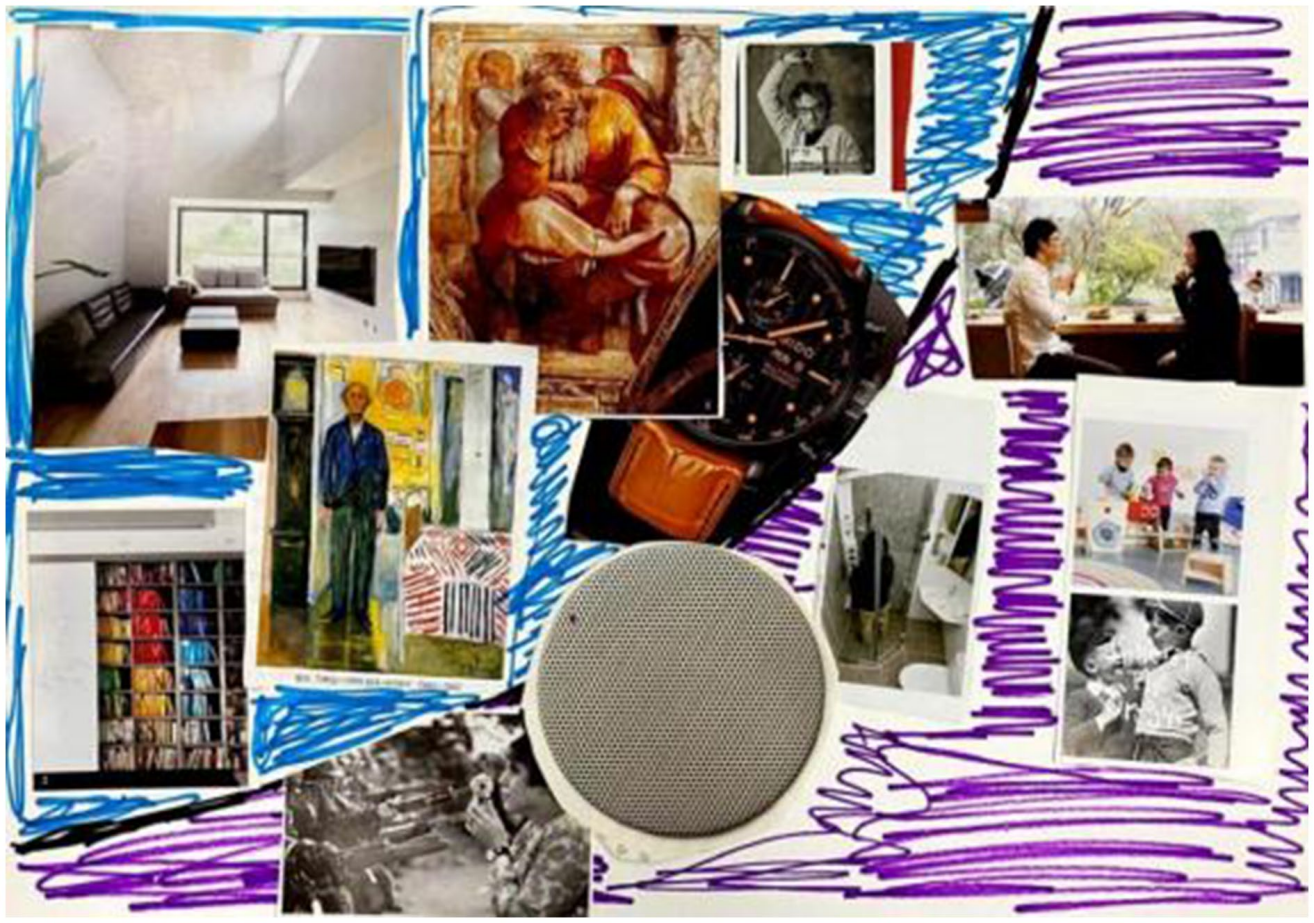
“Light and shadow” by Liu. Reproduced with permission.

Participants acquired the tools to understand their hearts, learning how to objectively review and organize themselves while exploring and improving their shortcomings. This gave them a deeper understanding of themselves and allowed them to master new methods of self-understanding. For instance, Liu learned how to examine himself objectively (Figure 2):

The best part is that I learned to look at myself. I use purple to indicate anxiety, depression, and escape. The blue part is the positive part, which represents rationality. I learned to look at myself dialectically.

**Figure 3:**
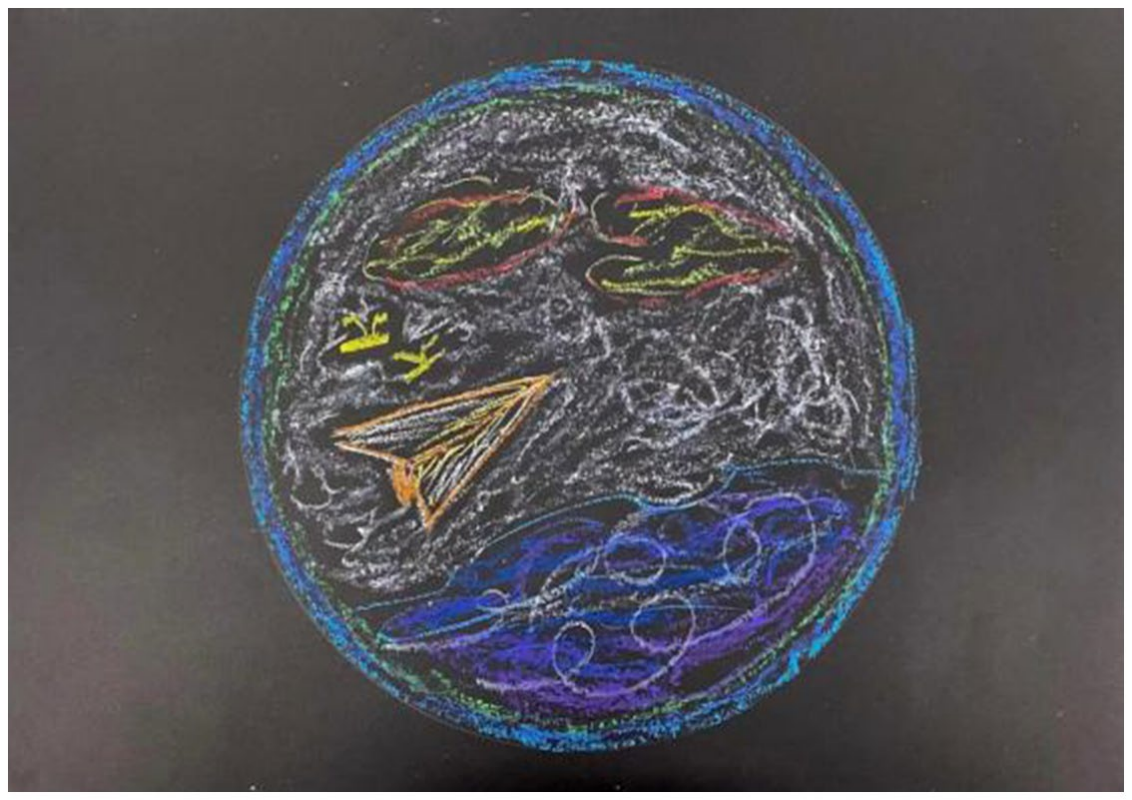
“Fly” by Liu. Reproduced with permission.

Shi “combs his past step by step, which is a very meaningful thing.” While adjusting himself, he “gives himself some strength.” Xie said that his mentality and thinking mode changed, and he was able to recognize problems and improve them. Wang developed a better understanding of herself through her art work, saying that “It can make me know myself better.” Li shared that he “discovered me and dug into myself” by painting. “This is a good way of (gaining) self-insight.” Liu liked talking with the therapist and learning about himself through the therapist’s analysis (Figure 3):

The therapist analyzed it, and his understanding of me was very accurate and magical. Drawing a paper airplane, the therapist said, “you are not a paper airplane; in fact, you have a lot of strength and resources, so you should make good use of it.” After the therapist dissected my work, I found that it virtually expressed my thoughts.

#### 3.2.3. Channels of self-expression

Participants found a channel for self-expression, learning to express themselves actively and openly during the sessions. Xie learned to “learn from other people’s experiences” to help their self-expression in English when their oral skills were insufficient. Shi said that he adjusted his mentality in time, admitted that “his works are inner expressions worth seeing,” and realized that he should focus on artistic expression and symbolism, rather than on the aesthetics of his works. Shi learned a new and safe way of expressing himself. Wang talked about her experience of learning to express herself: “I learned to express myself and open myself up. I will share my thoughts, opinions, and experiences with my friends.” Liu said that he experienced “a new and safe way of self-expression” through the therapeutic activities.

### 3.3. Theme 3: healing power of the mandala

#### 3.3.1. Relief in the mandala

Participants learned how mandalas could be a new tool to reduce stress, anxiety, and depression. Wang found that her inner repressed emotions were relieved: “Activities can not only relieve academic pressure but also relieve language pressure.” Li learned how new ways of mandala painting could allow him to vent his repressed emotions and “decompress himself.” Gong noticed group members becoming more comfortable and saw the mandala’s power to “get rid of the uneasy state” and “eliminate negative emotions.” Li said, “Depression is eliminated through activities.”

#### 3.3.2. The mandala as a safe space

**Figure 4:**
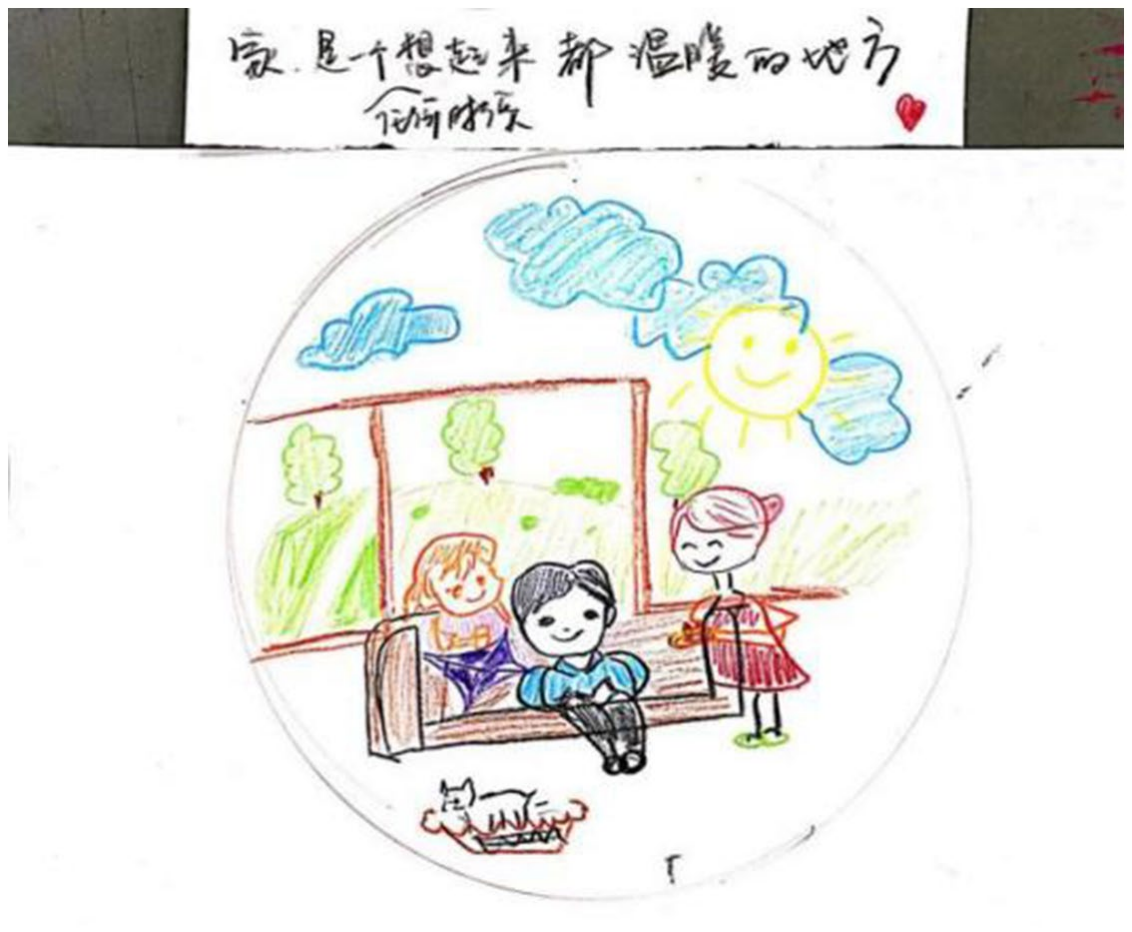
“Home is a warm place, whenever I think of it” by Wang. Reproduced with permission.

Participants, like Liu, gained a sense of security. Gong experienced reduced anxiety, saying that the “therapist shields me without prejudice, protects me, and makes me feel safe.” She also mentioned that “in Chinese culture, the circle is a symbol of balance”; thus, painting in the form of a mandala felt particularly comfortable to this group. Wang felt “the tolerance and warmth of the circle and felt a sense of security” during mandala painting. She said that painting in the circular form of a mandala made her feel warm, stable, and secure (Figure 4):

I like to draw in the circle. If you give me an empty piece of paper, I do not know what to do. In this circular range of the creation, I feel very practical, very safe. In the Chinese view, the circle is a symbol of safety and warmth. This circle is just like my home, giving me a sense of security.

#### 3.3.3. Leisure in the mandala

Participants found relaxation, which helped them release their negative emotions into art work. Liu said that “MBAT makes me feel more at ease and more gentle than other treatments, so I feel more acceptable. This kind of relaxation cannot be experienced by other treatment activities.” Xie said that he “no longer insists on things that cannot be changed, but is willing to accept other people’s voices” and “has a relaxed mood.” Li said that through treatment activities, “understanding the way of art creation is helpful to get psychological relaxation.”

#### 3.3.4. Satisfaction in the mandala

**Figure 5:**
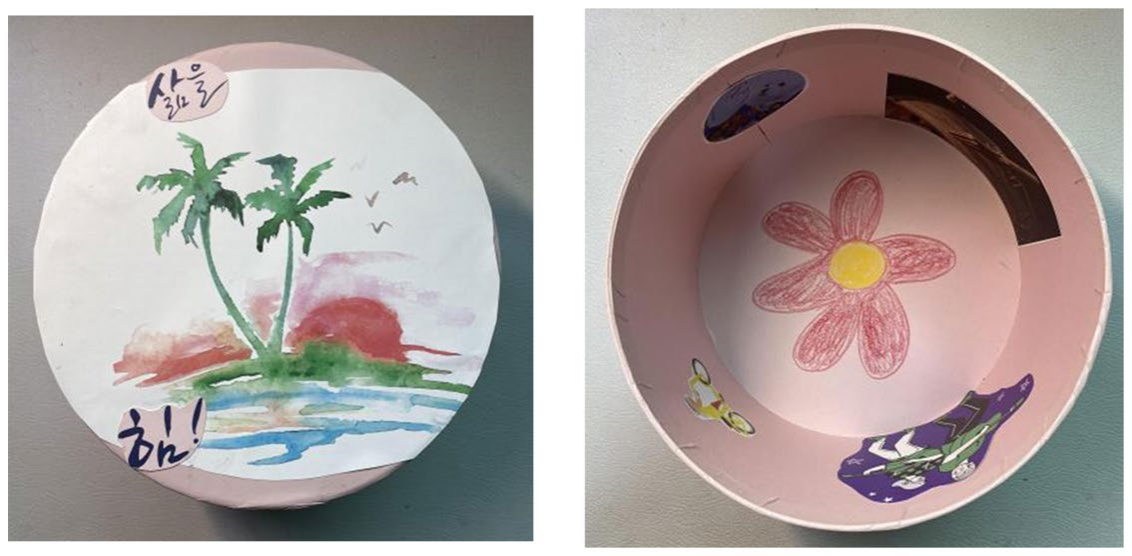
“Insight and integration” by Gong. Reproduced with permission.

Participants gained satisfaction by completing the art activities. Gong said that MBAT was a “compensation for the past,” satisfying her unfulfilled childhood wishes. Gong also talked about the satisfaction brought by the integrated works in the session (Figure 5).

This is the most perfect bike, guzheng (Chinese musical instrument), music, food, beach seagulls; everything I want. Particularly satisfactory. Finally, I returned, and I felt that the arrangement of heaven had come to an end for my mandala art activities.

**Figure 6:**
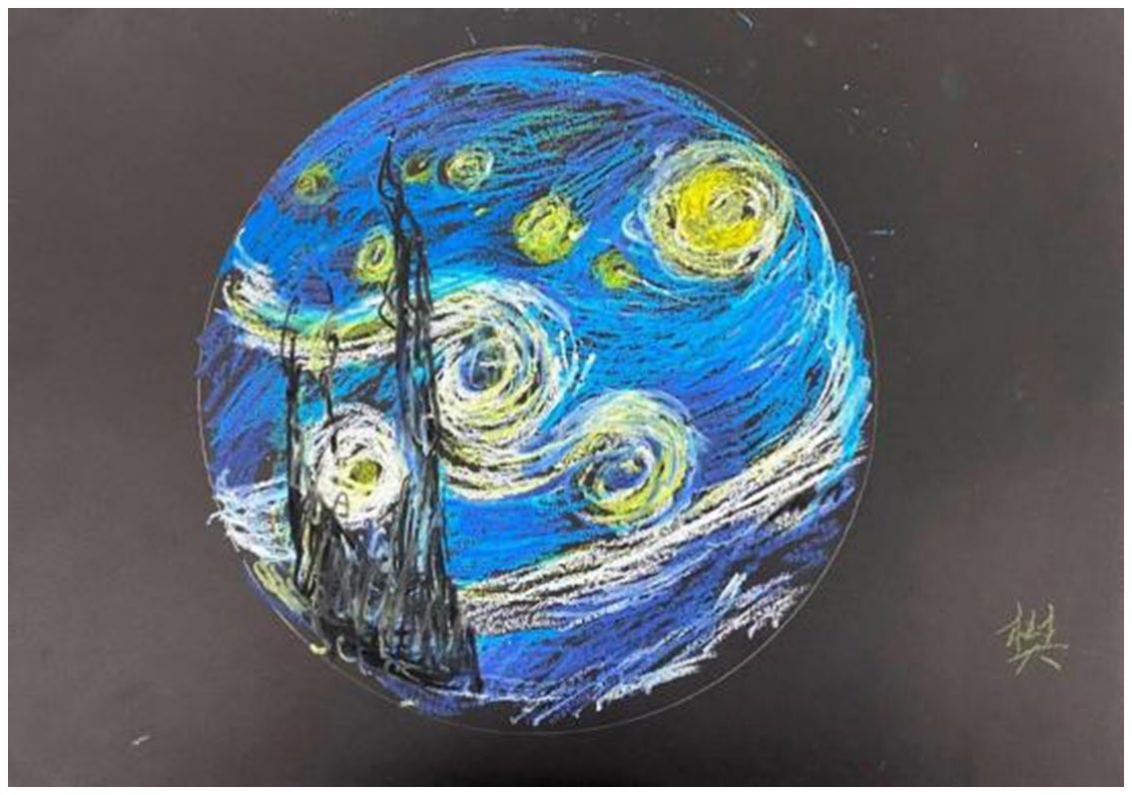
“Stars” by Han. Reproduced with permission.

Han experienced satisfaction when painting in the circle of a mandala (Figure 6).

Because my name means “the stars,” this starry sky can be combined into my name. And I like Van Gogh as a painter very much, and I am honored to express myself with his paintings. At that time, my companions all praised my good painting, and I felt very satisfied.

Participants found satisfaction through the demonstration, self-expression, and completion of their art works. Painting in a circular space gave participants a stronger feeling of completion. For many, this activity also met an unfulfilled childhood desire to love art.

#### 3.3.5. Confidence in the mandala

Participants gained confidence through the mandala group activities. Han said that he “accumulated a lot of energy” and “gained confidence and strength” in his work. Wang said that the activities were “of great help to confidence,” changing the mentality of self-distrust and helping her gain confidence in overcoming difficulties.

#### 3.3.6. Courage in the mandala

**Figure 7:**
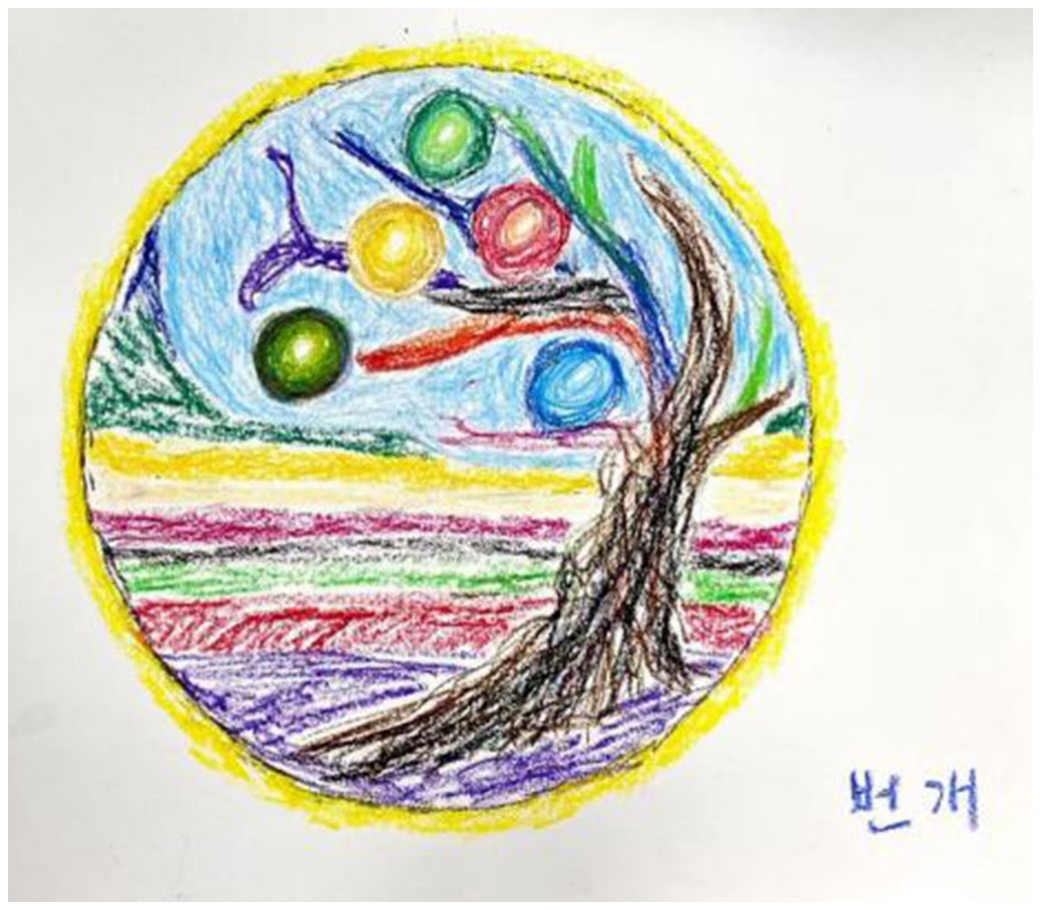
“Lightning” by Liu. Reproduced with permission.

Participants gained the courage and strength to face pain and overcome difficulties through sessions. Gong said that she gained the courage to face the trauma of her past. Liu noticed that he developed a better attitude, and that new ways of thinking gave him the strength to face and overcome difficulties. This was also reflected in his work (Figure 7):

I drew a circle of yellow light around me, something like lightning. This yellow light represents my courage to face difficulties, and it is a kind of power to help me achieve success. This work has given me the strength to face and overcome difficulties, and will play a positive role in my study abroad life and my future study and life.

#### 3.3.7. Vision in the mandala

Participants found that the group activities stimulated their longing for the future. Liu was able to clarify his future direction, and Gong was able to look forward to the future once again.

The most important thing about the whole activity is to have a little expectation for the future. I did not before, but now I seem to have a little expectation for the future. There are moments of harvest, healing brought by therapeutic activities, and my own reflection, which make me understand a lot. I do not worry about the past anymore. (Gong)

### 3.4. Theme 4: filling the inner space together

#### 3.4.1. Communicate sincerely with group members in the same situation

Participants communicated with their group members sincerely, deepening their mutual understanding. They felt a sense of understanding and support and were sincerely moved when they received notes of encouragement and blessing from group members during the event. Yun said that “mutual understanding among group members” was improved through sharing experiences. Gong was healed by the understanding and resonance of the members:

I used to think I was alone on the road, but now I know that there may be countless people like me around the dark. They got my signal. I feel that I have been understood by everyone, and I really want to cry. It is bringing me to tears.

**Figure 8:**
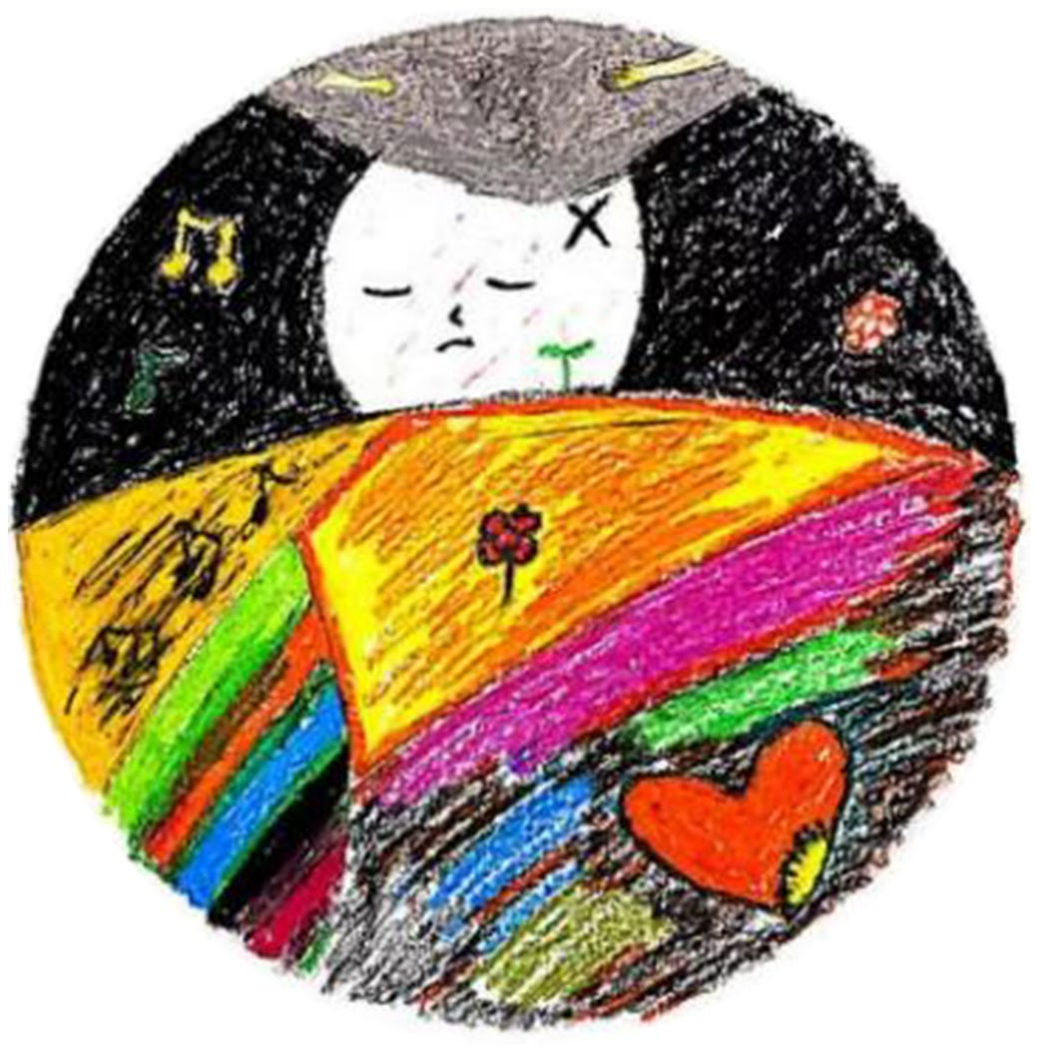
“The time of life” by Gong. Reproduced with permission.

Xie shared finding “solutions to difficulties” within the group through communication, “learning the views on things,” and receiving help through various experiences provided by group members. When Wang received letters from the group members, she felt “warm and moved” and “cured” by everyone’s encouragement. Gong communicated with the therapists by sharing his works, finding a new understanding of these works, and discovering a new way of detecting problems (Figure 8):

I was particularly impressed by a painting, which was the time when I described my life. When sharing, the therapist said, my portrait is to cover my past wounds with quilts; at that moment, I thought, “Oh, I can look at my life like this.” When I face this pain again, I look at past problems not from the perspective of the past, but from the perspective of the present.

Through sincere communication with others, the participants found a new understanding of their own works, gained new perspectives on problems, discovered new strength, and developed a better understanding of themselves. Mutual understanding among the group members was deepened through sincere exchange and communication.

#### 3.4.2. Emotional support from group members from the same cultural background

Participants found emotional support through the group. Xie confessed that “when you find yourself to be similar in the paintings of group members, you will resonate and influence each other.” The group members felt recognition and mutual support, which enhanced the trust between them. Gong was recognized and supported by group members through participation in group activities, feeling “that their creation is very meaningful.” Wang said that, through the sharing and unfolding of works, participants “trust each other more” and develop mutual understanding. It is this kind of support and trust that makes participants’ relationships closer.

#### 3.4.3. Interpersonal interaction activity among compatriot group members

Interpersonal relationships within the group improved across the sessions, creating a positive impact on interpersonal relationships in life outside the group. Gong said, “In the process of treatment, we can understand others and be more tolerant of other people’s phenomena and voices.” Through group activities, Gong learned to abandon prejudice, understand others, change his attitude toward others, and tolerate others. Shi described his experience of trying to open himself up in group activities by saying, “it is very important to actively communicate with each other in group activities” and “enhance openness” in group therapy. Li said that “the skills of communication and interpersonal communication have improved [and] the fear of social life has been alleviated”; he learned how to “actively make friends.” This indicated that the desire to establish and maintain harmonious relations with others increased through MBAT. Overall, an improvement in interpersonal relationships was seen across the participants.

### 3.5. Theme 5: opportunities to understand art therapy

#### 3.5.1. Improve expression skills through unfamiliar opportunities

There had been some psychological discomfort with painting as an unfamiliar tool during the early days of the MBAT. In the process of the fine art of mandala, owing to a lack of painting skills and difficulty with image performance, participants experienced psychological anxiety and an inferiority complex. In addition, participants indicated that they felt bound when painting within the circle of the mandala. Although they felt psychological discomfort while painting, their techniques of art and imagery performance improved through the MBAT.

**Figure 9:**
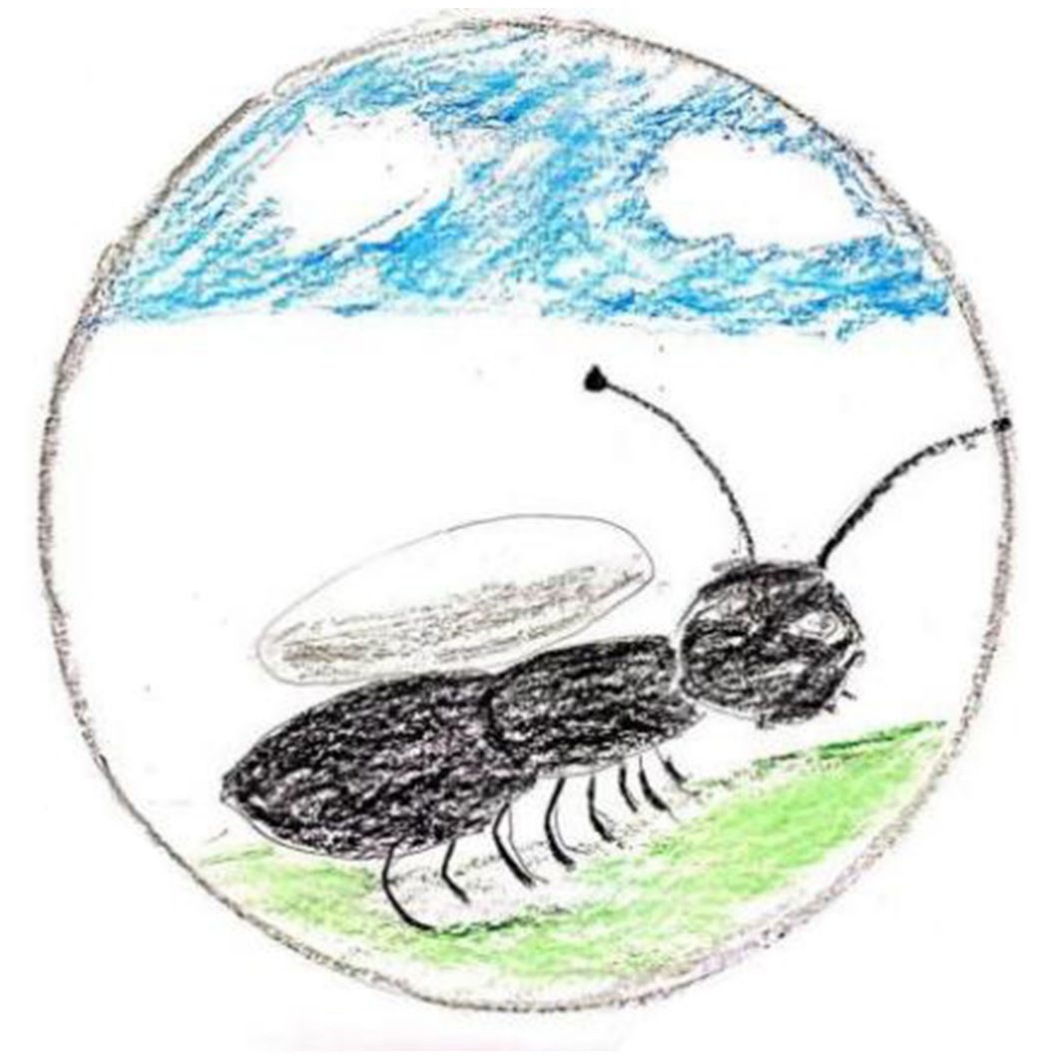
“Ant” by Shi. Reproduced with permission.

Gong felt that “painting is the biggest challenge” owing to his lack of skills, so it was “difficult to express his inner thoughts accurately.” Shi felt “confused” when he encountered “difficulties in expressing an image of paintings” in his work. Li said that “it was difficult to draw and caused anxiety.” Yun talked about feeling an “inferiority complex caused by insufficient painting skills” and even “discrimination by members in groups” in the creative process, owing to a lack of artistic ability. Shi also mentioned the sense of confinement or restriction created by the constraints of the mandala form (Figure 9).

In China, ants symbolize diligence and bustle. Shi expressed the tremendous pressure he felt in his busy life as an ant carrying a heavy burden. Shi believed that ants are trapped in circular rules, expressing his state of being bound and not free in life:

In the process of drawing, I thought of my current state. I have a lot of pressure; I feel like an ant, with a big burden on my back, leaving me out of breath. Drawing in the circular rule makes me feel bound and unable to move freely…there are many rules binding me.

Gong said that through the work process, his painting skills improved. Wang also mentioned the experience of improving her painting skills, “After MBAT, I feel that my drawing skills have been improved.” Most of the participants said that during the process of MBAT, they felt the difficulty of art creation, but through the mandala activity, they gained a new experience of improvement in their art ability.

#### 3.5.2. An insight into art therapy and mandala

Through the new experiences gained from MBAT, participants reported growing academically by participating in a group, especially as many of them were pursuing degrees related to expressive therapy. Han reported a deeper understanding of MBAT, which was of great help to his professional knowledge. This was an added benefit for participants in the physical education major, beyond personal growth. Wang shared that “in the art therapy activities, I have a certain understanding of art therapy and mandala.”

## 4. Discussion

We explored the experiences of Chinese students studying in South Korea, who participated in a group MBAT. The participants were challenged during the early stages of group activities, as they worked to build trust and overcome their fear of being vulnerable, alongside exposing their shortcomings in front of people they did not know. This was consistent with findings by Park and Park (2022). However, the participants gradually became comfortable communicating and deepened their understanding of group members. As they built a sense of trust, they were no longer afraid of other people’s views, and they found improved self-openness and positive self-expression. As in Kang (2021), participants with similar needs and conflicts can help solve each other’s problems through building mutual understanding and support, experiencing resonance from the group, thus promoting self-expression and openness. Therefore, for participants to benefit from group activities, they must first be guided to relax, let go of negative expectations, and build trust, before they can receive support and encouragement.

Through the intervention, participants focused on themselves, uncovering a better self-understanding and channel for self-expression. The participants enjoyed the process of painting in the mandala style, and experienced the value of focusing on the here and now. MBAT enables participants to understand the beauty of the painting process itself and find value in focusing on the present and self. The mandala’s circular form invites an obvious center, creating a point of focus (Riedel, 2002). It is thus clear that mandala art lends itself to creating self-awareness. These positive outcomes are consistent with similar studies (Kim and Kim, 2015; Kang, 2018; Park and Park, 2022).

The intervention helped participants understand their hearts. Riedel (2002) showed that through mandala art, people can easily gain self-awareness and input, and can better understand their internal situation through a sense of immersion. The participants in this study stated that they were able to examine themselves objectively through collective mandala fine arts psychotherapy activities. The mandala can thus be a channel for self-insight and self-discovery. This finding aligns with Kang’s (2018) study, in which the mandala helped participants find personal healing and discover themselves. This group of Chinese students studying in South Korea reported great value in understanding themselves better, exploring their needs, and identifying areas for growth.

The participants agreed that the mandala functioned as a channel for self-expression, consistent with Kim and Choi’s (2003) MBAT study. They also reported mastering new self-expression methods, consistent with Kim and Chun (2021).

Stress, anxiety, and depression were relieved through the sessions, and the participants came to consider mandala work as a new decompression method to promote mental peace and stability. A previous study (Judith, 1998) showed consistent results to our study, finding that MBAT was helpful in alleviating conflict and stress. Besides, studies have pointed out that depression can be overcome through MBAT (Sreetha et al., 2021). MBAT allows group members to verbalize stress (Lee, 2011); this could allow stressed, anxious, and depressed Chinese students in South Korea to make positive changes, bringing attention and healing to their inner selves.

The participants experienced the pleasure of creation and psychological relaxation. They gained satisfaction through the completion of their art works, improving their self-confidence, gaining the courage to face difficulties, building a sense of security, and clarifying future goals and expectations. The creative process can create an overall comfortable environment that brings positive emotional experiences. The mandala’s constrained form is special in that it creates both a safe haven for spiritual reconciliation and identity (Jung, 1973) and protects individuals from the outside world, creating a safe psychological space (Smitheman and Church, 1996).

Creating a mandala also helped the participants change their mentality and be more relaxed, while accepting the difficulties in their lives. This is consistent with Smitheman and Church (1996), who found that the constraints and features of the mandala circle contribute to relaxation. In addition, MBAT has been shown to promote psychological and physiological relaxation (Kim and Kim, 2015; Park and Park, 2022).

The participants had the opportunity to gain insight into other group members’ hearts and visualize their unconscious world; the art works they created promoted feelings of self-satisfaction and pride of achievement. When drawing the mandala patterns, participants felt a strong sense of perfection, consistent with Smitheman and Church (1996).

Moreover, the participants improved their confidence in overcoming difficulties and gained a high degree of self-confidence through sessions. This was consistent with Park and Park’s (2022) finding that group mandala promoted satisfaction, self-confidence, and positive self-awareness. Through this experience, participants could achieve their internal and external balance and regain self-confidence (Dahlke, 1999).

Creating complete mandala art works helped participants gain the courage to face difficulties and strength to overcome them. This was in line with the findings by Park and Park (2022). Kim and Choi (2003) also proposed MBAT to promote psychological preparation and the strength needed to accept difficulties among individuals. Mandala therapy has been proven helpful in activating hidden spiritual strength, adjusting people’s energy, and integrating their experience, promoting a positive attitude and helping overcome problems while gaining inner energy and strength (Moss, 2007; Babouchkina and Robbins, 2015).

Through the treatment activities, participants’ direction for the future was clearly defined, their soul was cured, and their expectations for the future were raised. This aligns with Moss’s (2007) finding that mandala art can help gain spiritual strength and find a sense of direction in both psychological and spiritual life. The participants also improved their sense of accomplishment and optimism about themselves and the future through the collective mandala art psychotherapy activities, in line with Park and Park (2022).

The participants had an expectation of healing when they joined the group. While they encountered emotional difficulties in this process, they also found courage and strength in facing difficulties. Participants expressed increased confidence, confirmed their inner strength, and reported having a better attitude toward overcoming difficulties in life.

The participants also reported receiving emotional support from group members through sincere communication, and interpersonal relationships improved through the group activities. This aligned with previous findings, whereby group mandala activities helped participants gain insight into distorted emotional and behavioral patterns, helping them become more energetic (Cha, 2012; Kim and Chun, 2015). These positive changes were brought about by taking part in activities together, sharing art works, engaging in communication, strengthening interactions with group members, learning to listen, accepting each other, overcoming isolation and shame, and discovering new energy (Park and Park, 2022).

The participants in this study were all Chinese students with similar cultural backgrounds, going through the similar experience of pursuing a doctoral degree abroad. This enhanced their ability to resonate with and support each other, build intimacy and trust, and generate a group identity. Shi shared the following:

In this activity, we are all compatriots from China. We have received the same education and culture in the process of growing up, so I have a natural sense of affinity and trust for them. Moreover, our current situation is the same. We all left our families and came to South Korea to study for a doctorate. We are facing the same difficulties, such as language barriers, missing our hometown, academic pressure, and so on. So, we understand each other’s plight, and we are more willing to share our inner feelings, hoping to help each other and grow together.

Through the process of drawing together, participants reported a positive emotional experience, which created a supportive atmosphere of belonging. This aligned with earlier findings by Park and Park (2022), who found that group mandala art promoted self-growth through the pleasure and benefits of collective participation.

Participants in the present study gained supportive relationships and a sense of identity, finding tolerance and understanding with the other group participants. This was consistent with Jun and Choi’s (1998) finding that group mandala psychotherapy had a positive impact on interpersonal relationships. As individuals build empathy, they are able to communicate more smoothly with other members, understand each other better, and care for each other. This improves interpersonal skills and improves one’s feelings toward both the self and others (Kim, 2012).

At first, the participants were uncomfortable painting a mandala because it was an unfamiliar skill; however, they ultimately developed the required artistic skills, which benefited them both academically and personally. When participants lacked the necessary artistic skills, they felt nervous, anxious, and inferior painting in front of others, fearing a negative evaluation from other group members. It is natural for people to avoid experiences that provoke anxiety (Newman et al., 2022). In learning something new, one must not only master the skill but also overcome fears and cognitive barriers (He, 2020). At the beginning of the event, although the participants felt psychologically uncomfortable with painting as an unknown tool, they mastered the ability and knowledge of art through actively experiencing mandala work, and confirmed an improvement in art expression technology and the deep understanding of mandala and art therapy. In sum, MBAT clearly benefited these Chinese doctoral students, providing them with a helpful tool to express their inner emotions while finding personal and academic growth.

It is worth noting that in the face of such positive findings, there are potential biases. As six of the participants were international students majoring in art and music, it may be easier for them to receive psychotherapy and be more familiar and open, perhaps making it easier to obtain positive results.

## 5. Conclusion

We found that Chinese doctoral students in South Korea worked through diverse emotional difficulties and built useful strengths through MBAT. They reported relieving their pain, challenging themselves, and expressing themselves better. Sharing pain in a supportive group gave them a sense of security and satisfaction, and improved their self-confidence and interpersonal relationships. MBAT provided a space in which they could paint their anxieties and navigate feelings of inferiority. Through the process of painting a mandala, they were guided to visit their inner self, reconnecting with the sense of order, achieving balance, and strengthening themselves. Drawing the mandala promoted self-awareness, self-expression, inner healing, and social ties. This safe psychological and emotional home gave participants a tool to express negative feelings, gain satisfaction, and build self-confidence, helping them generate the courage and strength they needed to face and look forward to the future.

However, this study had some limitations. The researchers were non-native English speakers. Therefore, while translating the direct quotations from the interviews, there is a possibility that the meaning was not captured accurately. Second, no follow-up interviews were conducted after the participants completed the eight art therapy sessions; therefore, the persistence of their meaningful experiences cannot be confirmed.

Regarding the future implications of this study, the researchers find huge value in conducting similar studies with pre- and post-assessments of depression, anxiety, and so on. Validating the clinical impact of MBAT could greatly increase the reach of such an intervention. The researchers recommend a subsequent continuous in-depth research protocol to provide various MBAT programs. If a long-term scheme of MBAT is designed and participants can fully experience the program, richer research results can be obtained. It is hoped that concluding this intervention will help participants complete their studies more effectively and achieve their ideal as well as future career goals, enabling them to ultimately return home safely with both personal and academic growth.

## Data availability statement

The original contributions presented in the study are included in the article/supplementary material, further inquiries can be directed to the corresponding author.

## Ethics statement

The studies involving humans were approved by Jeonju University, Korea, IRB was approved. The studies were conducted in accordance with the local legislation and institutional requirements. The participants provided their written informed consent to participate in this study. Written informed consent was obtained from the individual(s) for the publication of any potentially identifiable images or data included in this article.

## Author contributions

YM: Writing – original draft. KK: Supervision, Writing – review & editing.

## References

[ref1] Babouchkina A. Robbins S. J. (2015). Reducing negative mood through mandala creation: a randomized controlled trial. Art Ther. Alex. 32, 34–39. doi: 10.1080/07421656.2015.994428

[ref2] Cha H. (2012). Effect of group art therapy with mandala meditation on the brainwaves of the adult women and the control function of their frontal lobes. Korean J. Art Ther. 12, 73–91.

[ref3] Choi H. S. Choi W. S. (2012). The effects of group art therapy using mandalas on improving the emotional intelligence of children. Korean J. Art Ther. 19, 291–318. doi: 10.35594/kata.2012.19.2.006

[ref4] Dahlke R. (1999). Arbeitsbuch zur mandala-therapie [mandala therapy workbook]. Munchen: Schirner Verlag.

[ref5] Frederick W. J. (2005). Phenomenological research methods for counseling psychology. J. Couns. Psychol. 52, 167–177. doi: 10.1037/0022-0167.52.2.167

[ref6] Giorgi A. (2009). A descriptive phenomenological method in psychology: a modified Husserlian approach. Pittsburgh: Duquesne University Press.

[ref7] Girija K. S. Adlin P. J. R. (2018). The healing nature of mandala magic. Int. J. Nurs. Educ. Res. 6, 281–282. doi: 10.5958/2454-2660.2018.00066.2

[ref8] He X. (2020). Analysis and study of the easy problems and solutions in music learning. North. Music 2, 223–224.

[ref9] Ismail H. H. Yang Z. (2003). Cultural and gender differences in perceiving stressors: A cross-cultural investigation of African and western students in Chinese colleges. Stress. Health 19, 217–225. doi: 10.1002/smi.978

[ref10] Janesick V. J. (2004). Stretching exercises for qualitative researchers 2nd Ed. London: Sage. 10.7748/nr.12.1.87.s7 28718739

[ref11] Jin L. Yang E. Zamudio G. (2022). Self-determined motivation, acculturation, academic burnout, and psychosocial well-being of Chinese international students in South Korea. Couns. Psychol. Q. 35, 1–18. doi: 10.1080/09515070.2021.1887084

[ref12] Judith A. R. (1998). Art therapy: an introduction. NC Lillington: Edwards Brothers.

[ref13] Jun M. Choi E. (1998). The effectiveness of group art therapy in improving adolescents’ self-esteem and social-adjustment ability. Korean J. Art Ther. 5, 75–90.

[ref14] Jung C. G. (1973). Mandala symbolism. New Jersey: Princeton University Press.

[ref15] Kang D. Y. (2018). Study on archetype and mandala-symbol. J. Fish. Mar. Sci. Educ. 30, 86–97. doi: 10.13000/JFMSE.2018.02.30.1.86

[ref16] Kang C. (2021). The impact of collective art therapy on the LEARNED lethargy. Regulations 6, 10–18. doi: 10.22471/Regulations.2021.6.3.10

[ref17] Kim J. (2012). Effects of the cooperative mandala to improve empathy and peer relations of institutionalized children. J. Korean Child. Art Soc. 17, 19–42.

[ref18] Kim H. (2015). A study on the characteristics of Korean pronunciation for Chinese speaking learners of all levels: based on nasalization and lateralization [master’s thesis]. Gyeongsan: Yeungnam University.

[ref19] Kim D. Y. Choi Y. J. (2003). A study on the effect of mandala art therapy on depression and self-expression of stroke patient. J. Rehabil. Psychol. 32, 167–173.

[ref20] Kim J. Chun S. (2015). Effects of mandala-centered group art therapy on emotional intelligence and sociality of younger elementary school children. Korean J. Art Ther. 22, 1787–1806. doi: 10.35594/kata.2015.22.6.011

[ref21] Kim E. Y. Chun S. Y. (2021). The effect of mandala centered group art therapy on self-esteem and self-expression of schizophrenic patients. J. Korean Soc. Wellness 16, 184–190. doi: 10.21097/ksw.2021.02.16.1.184

[ref22] Kim S. H. Kim K. U. (2015). The effect of game and mandala on the attention of school-aged children. J. Dig. Converg. 13, 525–533. doi: 10.14400/JDC.2015.13.8.525

[ref23] Kim H. Kim S. Choe K. Kim J. S. (2018). Effects of mandala art therapy on subjective well-being, resilience, and hope in psychiatric inpatients. Arch. Psychiatr. Nurs. 32, 167–173. doi: 10.1016/j.apnu.2017.08.008, PMID: 29579508

[ref24] Ko J. Y. Kim Y. J. (2020). The characteristics of responses to PITR (person-in-the-rain) assessment based on the level of depression, acculturative stress of Chinese international students. Korean J. Arts Ther. 20, 101–123. doi: 10.18253/kart.2020.20.1.05

[ref25] Lan S. (2021). Finding a chulu (way out): rural-origin Chinese students studying abroad in South Korea. Pac. Aff. 94, 661–681. doi: 10.5509/2021944661

[ref26] Lee E. S. (2011). A single case study of mandala art therapy for middle aged women’s self-esteem and ways of stress coping. Korean J. Art Ther. 18, 1279–1302. doi: 10.35594/kata.2011.18.6.006

[ref27] Lee K. A. Byeongkug S. (2010). The effects of mandala group art therapy on shizophrenes’ self-esteem. Korean J. Art Ther. 17, 1431–1446. doi: 10.35594/kata.2010.17.6.007

[ref28] Lewis S. (2015). Qualitative inquiry and research design: choosing among five approaches. Health Promot. Pract. 16, 473–475. doi: 10.1177/1524839915580941

[ref29] Lincoln Y. S. Guba E. G. (1985). Naturalistic inquiry. London: Sage.

[ref30] Mabry L. (1998). “Case study methods” in Advances in educational productivity. eds. Reynolds A. J. Walberg H. J. (New York: JAI Press), 155–170.

[ref31] Malchiodi C. A. (2006). The art therapy sourcebook. New York: McGraw-Hill Education.

[ref32] Ministry of Education of Korea (2021). Basic statistical results of education in 2021. Seoul: Educational Statistics Service.

[ref33] Moss R. (2007). The mandala of being: Discovering the power of awareness. Novoto: New World Library.

[ref34] Moustakas C. (1994). Phenomenological research methods. London: Sage.

[ref35] Newman M. G. Rackoff G. N. Zhu Y. Kim H. (2022). A transdiagnostic evaluation of contrast avoidance across generalized anxiety disorder, major depressive disorder, and social anxiety disorder. J. Anxiety Disord. 93:102662. doi: 10.1016/j.janxdis.2022.102662, PMID: 36565682 PMC10080671

[ref36] Paley J. (2016). Phenomenology as qualitative research. London: Taylor and Francis.

[ref37] Park Y. S. (2014). The effects of the mandala group art therapy on the crisis adolescent’s psychological wellness and adaptation at vocational education high schools. Korean Psychol. Serv. Assoc. 6, 53–74.

[ref38] Park S. Y. Park K. H. (2022). Effects of group mandala art therapy on the self-expression, self-esteem and resilience of women with schizophrenia. J. Arts Psychother. 18, 1–28. doi: 10.32451/KJOAPS.2022.18.3.001

[ref39] Riedel I. (2002). Tiefenpsychologische dentung von kries, kreuz, dreirck, quadrat, spirale and mandala [depth psychological definitions of the circle, cross, triangle, square, spiral and mandala]. Seoul: Papier.

[ref40] Smitheman B. V. Church R. R. (1996). Mandala drawing: facilitating creative growth in children with ADD or ADHD. Art Ther. (Alex). 13, 252–260. doi: 10.1080/07421656.1996.10759233

[ref41] Spall S. (1998). Peer debriefing in qualitative research: emerging operational models. Qual. Inq. 4, 280–292. doi: 10.1177/107780049800400208

[ref42] Sreetha P. Dhanya G. A. Valsan N. (2021). Effect of coloring mandala art on depression among elderly persons. Int. J. Nurs. Educ. Res. 9, 185–188. doi: 10.5958/2454-2660.2021.00045.4

[ref43] Stout G. F. (2014). Analytic psychology. London: Taylor and Francis.

[ref44] Tian X. (2020). Influence of international student academic sentiment on learning immersion and school identity in COVID-19 distance education: Focusing on the mode rating effect of cultural adaptability of international students. [Unpublished master’s thesis]. Xinjiang: Xijiang University.

[ref45] Van Lith T. (2016). Art therapy in mental health: A systematic review of approaches and practices. Arts Psychother. 47, 9–22. doi: 10.1016/j.aip.2015.09.003

[ref46] Van Manen M. (1990). Beyond assumptions: shifting the limits of action research. Theory Pract. 29, 152–157. doi: 10.1080/00405849009543448

[ref47] Wang S. B. (2019). A comparative study on group privacy awareness in Chinese culture. [master’s thesis]. Beijing: Yanshan University.

[ref48] Yalom I. D. (2020). The theory and practice of group psychotherapy. 6th Edn. New York: Basic Books.

[ref49] Yang Y. J. Lee K. M. (2010). Effects of short-term group art therapy in decreasing accumulative stress in foreign workers. J. Arts Psychother. 6, 1–20.

[ref50] Yu G. Y. (2021). A study on the experience of bereaved parents when six years has passed after the Sewol ferry disaster: Using Giorgi’s phenomenological method [Unpublished Doctoral dissertation]. Gwangju: Chonnam National University.

[ref51] Zhang Y. (2015). The relationship comparison of acculturation types, stress coping styles, and psychological well-being of Chinese students studying in Korea and Korean students studying in China. [Unpublished Doctoral dissertation]. Mokpo, Jeollanam-do: Mokpo National University.

[ref52] Zhang C. Shi L. Tian T. Zhou Z. Peng X. Shen Y.. (2022). Associations between academic stress and depressive symptoms mediated by anxiety symptoms and hopelessness among Chinese college students. Psychol. Res. Behav. Manag. 15, 547–556. doi: 10.2147/PRBM.S353778, PMID: 35282002 PMC8906854

[ref53] Zhao L. F. Lee D. (2018). Moderating effects of ego-defense mechanism in the relationship between acculturative stress and depression in a sample of Chinese college students. Korean J. Counsel. 19, 141–158. doi: 10.15703/kjc.19.3.201806.141

